# Hospital efficiency assessment: a systematic review of DEA and hybrid DEA–machine learning approaches

**DOI:** 10.3389/fmedt.2026.1820803

**Published:** 2026-06-10

**Authors:** Nesrin Alkan, Ubeyde Kaan Koluk, Bilal Baris Alkan, Alper Sinan

**Affiliations:** 1 Faculty of Economics and Administrative Sciences, Akdeniz University, Antalya, Türkiye; 2 Department of Educational Sciences, Akdeniz University, Antalya, Türkiye

**Keywords:** data envelopment analysis, decision-making, hospital efficiency, machine learning, multiple correspondence analysis, systematic review

## Abstract

The increasing complexity of healthcare systems and growing resource constraints have made hospital efficiency assessment central to healthcare management and policy. Data Envelopment Analysis (DEA) has been widely used to evaluate hospital efficiency due to its ability to accommodate multiple input and output structures. However, classical DEA applications provide limited support for explaining efficiency scores, generating predictions, and informing managerial decision-making. This study systematically reviews hospital efficiency studies published between 2020 and 2025 to examine the use and limitations of DEA and to identify approaches integrating DEA with machine learning (ML). To more comprehensively demonstrate the methodological diversity of DEA-machine learning integration, studies from non-healthcare fields have also been included in the comparative analysis. Following PRISMA guidelines, standalone DEA and DEA–ML studies were analysed and coded across methodological dimensions. Multiple Correspondence Analysis was applied to identify dominant methodological configurations and emerging patterns in the literature. Findings indicate that CCR and BCC models remain prevalent, with human resources and financial indicators as common inputs and service delivery measures as outputs. DEA–ML research largely relies on two-stage structures focused on classification and prediction. The results highlight methodological gaps and the potential of more explainable and decision-support-oriented DEA–ML approaches to enhance benchmarking and resource allocation in hospital efficiency management.

## 1 Introduction

Health systems, comprising organizations, resources, and activities aimed at protecting, improving, and maintaining health, are among the key indicators of a country's social welfare and development levels. Every country is responsible for improving and protecting the health of its population, and effectively fulfilling this responsibility depends on the provision of effective prevention, protection, and treatment services (1). In this context, evaluating the performance of health systems emerges as a critical requirement for the efficient use of limited resources, improving service quality, and enhancing health outcomes. However, due to high-cost infrastructure investments, a large and heterogeneous workforce, rapidly changing technologies, and fluctuating service demand, the health sector remains one of the areas where performance and effectiveness analyses are conducted in the most complex manner.

Performance evaluations conducted at different levels of health systems serve as an important decision-support tool for policymakers in improving resource allocation, for managers in optimizing operational processes, and for ensuring quality and equity in service delivery. In particular, efficiency analyses at the hospital level play a strategic role in ensuring financial sustainability, improving service quality, and strengthening performance management systems (2). In this context, the concept of efficiency, a fundamental economic principle expressing the production of the highest output with available inputs, is widely used in the healthcare literature (3, 4). The foundations of modern productivity measurement were laid by Farrell (5), and his framework continues to guide contemporary performance analysis in healthcare.

Data Envelopment Analysis (DEA) has been one of the most widely used methods in the literature for many years in measuring hospital efficiency, due to its ability to simultaneously address multiple input and output structures. Different DEA approaches, primarily the CCR (6) and BCC (7) models, have made significant contributions to measuring the relative technical efficiency of hospitals. However, a significant portion of DEA-based studies in the hospital efficiency literature is limited to calculating and comparing efficiency scores; they offer a limited analytical framework for explaining the reasons for these scores, generating future predictions, and developing achievable improvement goals. Furthermore, factors such as data heterogeneity, measurement errors, and insufficient representation of the quality dimension further limit the explanatory power of classical DEA models.

In recent years, the increasing complexity of healthcare systems and the rise in the volume and diversity of data have made these limitations more visible, leading researchers to hybrid approaches that integrate DEA with Machine Learning (ML) methods. Machine learning, a subfield of artificial intelligence (AI), focuses on developing algorithms that enable computers to learn from data without explicit programming (8, 9). ML methods, which emerged from early AI research in the 1950s (10, 11), are designed to identify patterns, classify information, generate predictions, and support decision-making in complex systems (12, 13).

DEA-machine learning integration should be considered not only as a technical extension to overcome the limitations of DEA, but also within the context of the evolution of decision support systems and data-driven analytics approaches. The literature emphasizes that decision support systems have evolved over time from descriptive and explanatory analyses to predictive and prescriptive analytical approaches (14). In this evolutionary process, the prediction, classification, and variable significance determination capabilities offered by machine learning methods are significantly expanding the scope and decision support capacity of analytical models (15). In this context, the efficiency measurement and comparative analysis capabilities provided by DEA, combined with the advanced analytical capacity offered by machine learning methods, create a more comprehensive decision support structure. Furthermore, the recently prominent Explainable AI approaches play a significant role in decision support processes by increasing the interpretability of machine learning models (16). Therefore, DEA-machine learning integration can be considered a natural part of the evolution of analytical methods.

Within this theoretical framework, DEA-machine learning integration offers significant analytical advantages in concrete applications. DEA-ML integration offers more flexible and powerful analytical capabilities compared to classical DEA in areas such as estimating efficiency scores, classifying hospitals based on their similarities, determining variable importance, and setting explainability and targets. However, the limited number of studies in the literature that combine DEA and machine learning methods in the context of hospital efficiency makes it difficult to comprehensively understand the methodological structure of this integration by focusing solely on the healthcare field. Therefore, in order to better understand the methodological diversity and analytical potential of DEA-ML integration, this study also includes DEA-ML studies conducted in different application areas outside the healthcare sector. This choice is consistent with the cross-domain methodological transferability approach in the literature, which refers to the adaptability of analytical methods across different problem contexts. Machine learning plays a role not only in prediction but also in model and theory development (17); DEA, on the other hand, is a domain-independent method applicable in different sectors (18, 19). Therefore, examining the applications of these methods in different fields is methodologically meaningful. Rather than expanding the scope, this approach aims to reveal the purposes of DEA-machine learning integration in different contexts, the data structures and analytical strategies used, and to provide a framework for how these can be adapted to hospital efficiency analyses. The purpose of including non-healthcare studies is to comparatively demonstrate how DEA and machine learning methods are used together in different fields. These studies are not used to directly generate hospital efficiency results, but rather to support the interpretation of health findings and to provide researchers with a methodological frame of reference.

Accordingly, this study systematically examines how DEA is used in hospital efficiency studies published between 2020 and 2025 and the limitations of this approach; then, it evaluates, from a holistic perspective, how these limitations are attempted to be overcome through methodological approaches based on DEA-ML integration studies. This systematic review, conducted in line with the PRISMA guidelines, aims to provide a critical and guiding contribution to the literature by revealing the methodological transformation from classical DEA practices to DEA-ML integration.

In this context, multiple correspondence analysis was applied to go beyond merely describing the methodological characteristics of the articles examined in the study and to reveal the implicit relationships in the literature in a deeper way. Correspondence analysis is an exploratory multivariate technique that aims to represent the relationships between categorical variables in a low-dimensional geometric space based on chi-square distances (20). Thus, it goes beyond simply revealing which methods are used how frequently in the literature, enabling the discovery of structural relationships and implicit methodological patterns among the studies. While traditional narrative-based syntheses and frequency tables are limited to describing the methodological diversity in the literature, multiple correspondence analysis allows for a multidimensional representation of the literature by simultaneously considering multiple categorical variables. In this way, the natural clustering of studies sharing similar methodological approaches can be visually determined; dominant research paradigms and marginal or innovative methodological orientations can be more clearly distinguished.

In the multiple correspondence analysis applied within the scope of this study, the functional roles of machine learning algorithms within DEA-based analytical frameworks (such as classification, prediction, variable significance determination, target setting, and explainability) were emphasized, rather than their technical details. The studies examined were evaluated together in terms of research objectives, DEA-ML integration directions, application areas, and data structures, aiming to map methodological similarities and divergences that are difficult to reveal with classical systematic review approaches. This approach reveals the methodological transitions, hybridization patterns, and research directions that are still in the maturing stage in the literature, evolving from DEA to machine learning, in an analytical and interpretable way. Therefore, multiple correspondence analysis significantly strengthens the theoretical depth and methodological originality of the study by transforming the scattered and heterogeneous DEA-ML literature into a holistic methodological map.

In this context, the study aims not only to describe the literature on the combined use of DEA and ML methods, but also to systematically analyze the methodological structure and analytical logic behind this integration. Accordingly, the research questions of the study are structured as follows:

RQ1: In DEA-based studies evaluating hospital efficiency, which input and output variables are predominantly used, and what trends have these variable selections shown over time?

RQ2: In the literature, within which methodological forms and analytical approaches is the integration of DEA and ML methods applied?

RQ3: In which sectors, primarily healthcare, are DEA-ML integrations applied; which data types (cross-sectional, panel, big data, etc.) and analytical environments are preferred in these applications?

RQ4: How can the methodological diversity observed in the DEA-ML literature be systematically classified within the context of dominant, transitional, and innovative research clusters?

The multiple correspondence analysis applied in seeking answers to these research questions allows for the answer not only to the question of “which method has been used how often?”, but also to more in-depth questions such as which methods have been used together for what purposes, in what contexts, and in what analytical roles, how these combinations are clustered in the literature, and through what patterns the observed methodological transformation from DEA to machine learning has occurred. Thus, this study aims to go beyond mapping the current state of DEA-ML integration and to provide a methodological classification framework for the hospital efficiency literature and an analytical roadmap to guide future research.

## 2 Materials and methods

### 2.1 Study design

**Figure 1:**
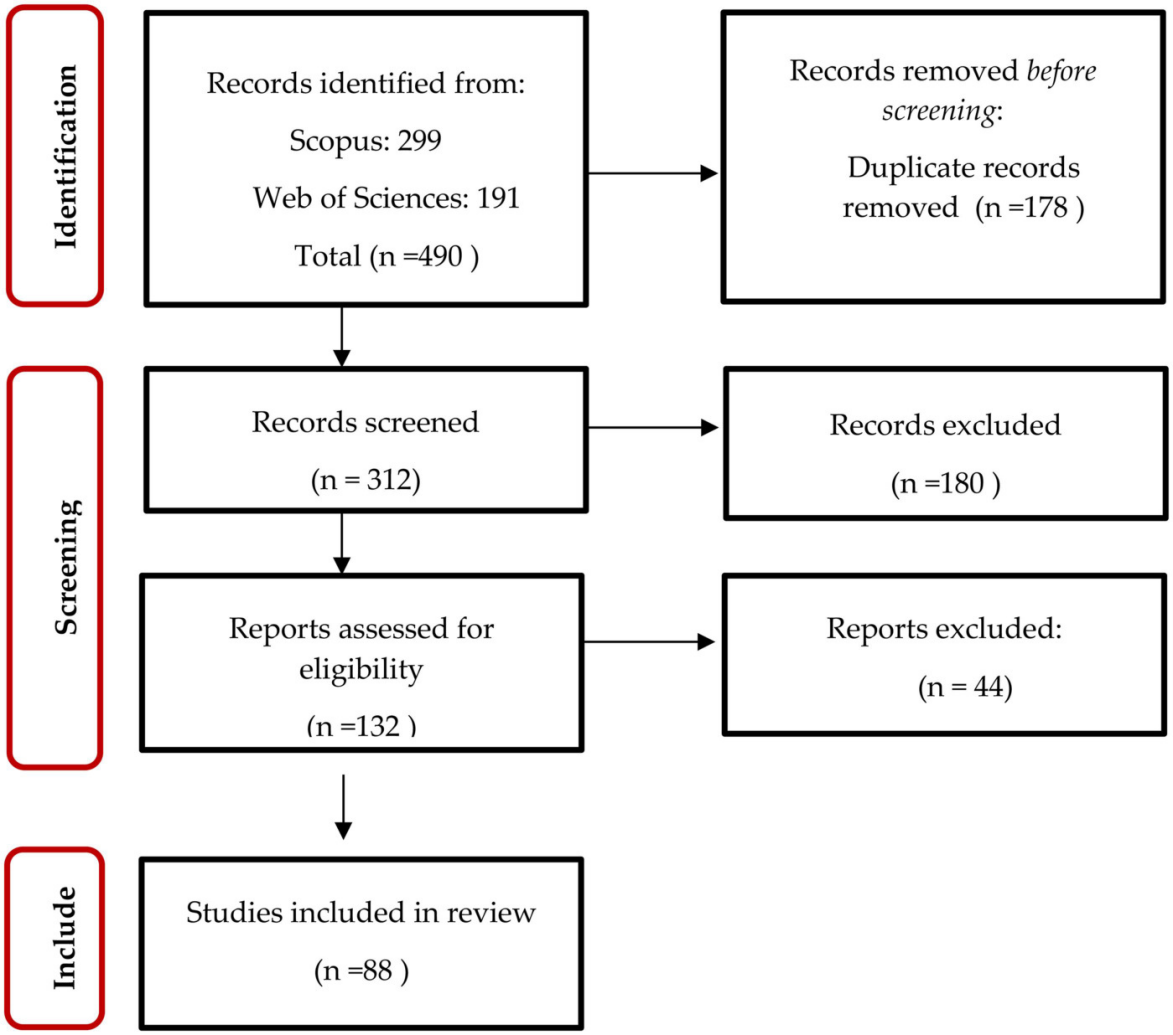
PRISMA flowchart.

This systematic review was conducted in accordance with the Preferred Reporting Items for Systematic Reviews and Meta-Analyses (PRISMA) guidelines (21, 22). The PRISMA framework ensures a transparent and replicable process for the identification, screening, and synthesis of relevant studies. The review process comprised four main stages: identification, screening, eligibility assessment, and inclusion. The overall workflow of the review is presented in Figure 1.

The systematic review framework was structured based on the PICOS criteria. The population (P) consisted of hospitals and healthcare systems evaluated in efficiency studies. The intervention (I) included the application of Data Envelopment Analysis (DEA) and DEA–machine learning hybrid approaches. The comparator (C) involved different DEA model specifications or alternative analytical approaches used for performance evaluation. The outcomes (O) focused on efficiency scores, performance assessment, and benchmarking results. The study design (S) was limited to empirical studies employing quantitative data and analytical methods. This framework guided the study selection, data extraction, and synthesis processes.

### 2.2 Search strategy

In this study, the literature review and study selection process was conducted systematically in accordance with the PRISMA (Preferred Reporting Items for Systematic Reviews and Meta-Analyses) guidelines. The literature search was performed using two separate keyword groups, covering the period 2020–2025 in the Web of Science (WoS) and Scopus databases.

The literature search was conducted in two stages using two separate keyword groups in the Web of Science and Scopus databases. In the first stage, searches using the keywords “hospital efficiency” AND “data envelopment analysis” yielded 61 publications in the Web of Science database and 67 in the Scopus database. In the second stage, using the keywords “data envelopment analysis” AND “machine learning,” 130 records were identified in the Web of Science database and 232 in the Scopus database. The number of records obtained for each stage was documented separately. The final literature search was conducted on December 30, 2025, and only studies published in English were included in the review. After eliminating overlaps and duplicate records between databases, the remaining studies were subjected to a preliminary review at the title and abstract level. Publications outside the scope of the research were eliminated at this stage; studies deemed suitable were then subjected to full-text suitability evaluation. The screening and study selection process was conducted independently by two researchers. Title-abstract screening and full-text evaluations were performed separately, and any disagreements were resolved through discussion until consensus was reached. As a result of the full-text review, publications whose methodological framework, data structure, or analytical approach did not align with the study's objectives were excluded. At the end of this elimination process, a total of 88 articles were ultimately selected for inclusion in the study. Of these studies, 40 focus solely on DEA-based efficacy analyses in the context of hospitals and healthcare services, while 48 focus on the integration of Data Envelopment Analysis with machine learning methods. In this context, the inclusion of DEA (machine learning) studies from non-healthcare sectors was a conscious methodological choice. These studies were not evaluated with the aim of generating direct inferences regarding hospital efficiency, but rather to reveal how DEA integration is structured in different application areas and with which data structures and analytical strategies it is used. This approach allows for a more comprehensive examination of methodological approaches that can be adapted to hospital efficiency analyses, going beyond the limited number of DEA studies in the healthcare field. All stages of the study selection process are visualized in detail using the PRISMA flowchart.

The main reason for limiting the research to the 2020–2025 period is that approaches based on the integration of DEA and machine learning methods have shown a significant increase and methodological diversification in studies aimed at evaluating hospital efficiency, especially in the post-2020 period. During this period, it is observed that machine learning algorithms for classification and prediction purposes are used more systematically within the DEA framework; and analytical purposes such as explainability, determining variable importance, and target setting have come to the forefront. Focusing on this time period allows the study to evaluate the current input-output structures and analytical approaches used in the hospital efficiency literature within a more consistent framework. The literature review was conducted without any country or institutional context restrictions**.**

### 2.3 Inclusion and exclusion criteria

Studies were included if they:

1. The publication must have taken place between January 2020 and December 2025; this period should reflect both the current trends in DEA-based applications in the hospital efficiency literature and the process in which DEA-machine learning integration became methodologically prominent.
2. The studies should be empirical studies that directly evaluate healthcare or hospital efficiency and use DEA as the primary analytical tool in effectiveness measurement.
3. The studies should include either hospital and healthcare applications where DEA is used alone, or DEA-based frameworks where it is integrated with or compared to ML or artificial intelligence-based methods.
4. The studies should not be limited to theoretical or conceptual discussions but should present empirical findings based on measurable input and output variables.

Studies with the following characteristics are excluded:

1. Publications whose full text is not accessible.
2. Purely conceptual or methodological studies that do not include an empirical application or provide data-based analysis.
3. Studies that do not include the DEA method as a primary analytical component or use DEA only as a secondary/auxiliary tool.

### 2.4 Selection and data extraction process

As shown in the PRISMA flowchart in Figure 1, a total of 490 records were obtained from database searches. After removing duplicate records, 312 studies were subjected to title and abstract level searches. At this stage, 180 records were eliminated because they were inconsistent with the study topic, did not address hospital efficiency with DEA, or did not include the integration of Data Envelopment Analysis and ML methods. The remaining 132 studies were included in the full-text review and examined in detail according to the eligibility criteria. As a result of the full-text review, 44 studies were excluded for reasons such as not including an empirical application, not using DEA as a primary analysis method, or being outside the scope of the study. Finally, a total of 88 studies that met the inclusion criteria were included in the systematic review.

## Results

As a result of the literature review conducted in accordance with the PRISMA guidelines, a total of 40 studies published between 2020 and 2025 that evaluated hospital efficiency using only the DEA method were included in this review. These studies were systematically examined to reveal the ways in which DEA is used and the preferred model types in the hospital efficiency literature. The basic characteristics of the examined studies are summarized in Table 1 in terms of author(s) and publication year, the DEA model used, and the type of hospital analyzed. The input and output variables used in the studies were evaluated separately and analyzed through frequency tables to reveal current trends.

**Table 1:** Summary of methodological characteristics of studies included in the systematic review.

Author(s) and Year	DEA model	Type of hospitals compared	Methodological approach	Purpose of second-stage analysis
Alatawi et al. (23)	CCR, BCC	91 MoH public hospitals in Saudi Arabia	DEA only	—
Ayiko et al. (24)	CCR, BCC	78 general hospitals in Uganda	DEA + Tobit	Determinants of efficiency
Barpanda and Sreekumar (25)	CCR	20 private hospitals in Kerala, India	DEA only	—
Berger et al. (26)	SBM, Super-efficiency DEA	120 public/non-profit hospitals in Austria	DEA only	—
Cho (27)	BCC	200 hospitals across 7 U.S. regions	DEA only	—
Küçük et al. (28)	BCC	669 MoH hospitals in Türkiye	DEA only	—
Mohamadi et al. (29)	CCR, MPI	Health systems of 36 countries	DEA only	—
Schneider et al. (30)	CCR, MPI	1,428 acute care hospitals in Germany	DEA + Double-bootstrap	Statistical validation of efficiency scores
Akula and Singh (31)	CCR, BCC	48 tertiary hospitals in Punjab, India	DEA only	—
Fumbwe et al. (32)	Dynamic DEA	133 public hospitals in Tanzania	DEA only	—
Han and Lee (33)	CCR, BCC	3,458 U.S. hospitals	DEA + Tobit	Determinants of efficiency
Peng et al. (34)	CCR, BCC	200 public hospitals in China	DEA + Tobit, PSM	Determinants of efficiency + Impact evaluation
Piubello Orsini et al. (35)	BCC, MPI	43 public hospitals in Italy	DEA + Tobit	Determinants of efficiency
Yeşilyurt et al. (36)	CCR, BCC	97 public hospitals in Türkiye	DEA + PSO	Optimization of efficiency scores
Yin et al. (37)	CCR, MPI	25 public hospitals in Wuhan	DEA only	—
Yue et al. (38)	SBM, MPI	61 TCM hospitals in China	DEA only	—
Chiu et al. (39)	Dynamic DEA	19 medical centers in Taiwan	DEA + Beta regression	Determinants of efficiency
Danilov (40)	BCC	56 state hospitals in Russia	DEA + Regression	Determinants of efficiency
Ghiyasi et al. (41)	Inverse DEA, DEA-R	130 public hospitals in Iran	DEA only	—
Guerra et al. (42)	CCR, BCC	50 hospitals in Brazil	DEA only	—
Yousefi et al. (43)	BCC	15 public hospitals in Western Iran	DEA + Tobit	Determinants of efficiency
Bağcı and Koçyiğit (44)	CCR, BCC, MPI	555 public hospitals in Türkiye	DEA + Statistical analysis	Descriptive analysis of efficiency trends
Dohmen et al. (45)	BCC, MPI	72 Dutch hospitals	DEA only	—
Farantos and Koutsoukis (46)	Window-DEA	Greek public hospitals	DEA only	—
Hajiagha et al. (47)	CCR	11 public hospitals in Iran	DEA + PCA + FA	Dimension reduction and model structuring
Lee et al. (48)	CCR, BCC, SBM	6,251 U.S. hospitals	DEA + Nonparametric tests	Group comparison and influencing factors
Sun et al. (49)	CCR, BCC	49 public hospitals in China	DEA + Tobit	Determinants of efficiency
Afonso et al. (50)	NDEA	27 hospitals in Portuguese NHS	DEA only	—
Chatterjee and Gangopadhyay (51)	CCR, BCC	25 hospitals in India	DEA + Tobit	Determinants of efficiency
Cinaroğlu (52)	BCC	Public hospitals in Türkiye (2005–2017)	DEA + PSM, DiD	Policy impact analysis
Li et al. (53)	BCC	434 U.S. hospitals	DEA + Semi-parametric	Determinants of efficiency
Lindaas et al. (54)	BCC, MPI	19 hospital trusts in Norway	DEA + OLS	Determinants of efficiency
Mitakos and Mpogiatzidis (55)	CCR, BCC	38 Greek hospitals (COVID-19)	DEA + AHP	Decision support/weighting
Nguyen et al. (56)	CCR, BCC	57 hospitals in Australia	DEA + DiD	Policy impact analysis
Wang et al. (57)	CCR, BCC	16 TCM hospitals in China	DEA only	—
Fourlopoulou et al. (58)	Cost DEA, MPI	109 Greek public hospitals	DEA only	—
Hadian et al. (59)	AP, Super-efficiency DEA, MPI	26 university hospitals in Iran	DEA only	—
Jiang et al. (60)	BCC	749 public hospitals in China	DEA + PSM, DID Tobit	Impact evaluation + Determinants of efficiency
Liao et al. (61)	CCR, BCC, SBM, MPI	TCM hospitals in China	DEA only	—
Shen et al. (62)	BCC	70 U.S. hospitals	DEA + Tobit & Logistic	Determinants + Classification

As summarized in Table 1, the BCC (Variable Returns to Scale) model was the most frequently applied (24 occurrences), indicating a preference for variable scale efficiency analyses over constant returns. The CCR (Constant Returns to Scale) model followed with 19 uses, while MPI (Malmquist Productivity Index) appeared eleven times, reflecting interest in dynamic productivity change analyses. Other DEA models such as SBM, NDEA, Super-efficiency DEA, and AP were used less frequently (Table 2). These patterns suggest methodological diversity across studies, although the predominance of BCC and CCR models indicates that most evaluations relied on traditional DEA frameworks.

**Table 2:** Frequency of DEA models used in the reviewed studies.

DEA model	Frequency (n)
BCC (Banker, Charnes and Cooper)	24
CCR (Constant Returns to Scale)	19
MPI (Malmquist Productivity Index)	11
SBM (Slacks-Based Measure)	4
Super-efficiency DEA	2
Dynamic DEA	2
NDEA (Network DEA)	1
AP (Additive Model)	1
Inverse DEA	1
DEA-R	1
Window-DEA	1
Cost DEA	1

Analysis of input variables (Table 3) revealed that number of beds (49), number of physicians (36), and number of nurses (34) were the most common indicators, emphasizing the importance of physical capacity and core human resources in efficiency measurement. Cost/expenditure (25) and staff count (25) were also frequently included, suggesting a focus on both technical and financial efficiency. While these variables were widely used, their definitions and measurement approaches varied across studies, which may limit direct comparability. Secondary inputs such as technological infrastructure and case complexity indicators were occasionally incorporated to capture contextual and operational differences, but these appeared inconsistently across the reviewed literature.

**Table 3:** Frequency of input variables used in the reviewed studies.

Category of input variable	Specific variable	Frequency (n)
Capacity	Number of beds/hospital beds	49
Core workforce	Number of physicians/doctors	36
Core workforce	Number of nurses	34
Cost/financial	Cost/expenditure/spending	25
Support workforce	Number of staff/other personnel/general health personnel	25
Infrastructure/equipment	Equipment/devices/infrastructure	5
Specialized capacity and technology	MRI/CT scanners, special beds, etc. (7 sub-inputs)	7
Operational and quality inputs	Case-mix index, length of stay, number of isolation rooms, services provided, etc. (8 sub-inputs)	8
Secondary financial inputs	Per capita expenditure, financial ratios, etc. (5 sub-inputs)	5

Regarding output variables (Table 4), outpatient volume (40), inpatient volume (37), and number of surgeries (31) were dominant, demonstrating a traditional focus on service volume and production capacity. Quality-related outputs such as infection rates (6) and mortality rates (9) were increasingly included, reflecting a shift toward comprehensive models integrating both efficiency and care quality. However, the inclusion of quality indicators was still limited relative to volume-based outputs, indicating that most studies prioritized production efficiency over holistic performance assessments. Additional measures such as bed occupancy rates, length of stay, and process-specific metrics were used in a subset of studies, contributing to methodological heterogeneity.

**Table 4:** Frequency of output variables used in the reviewed studies.

Category of output variable	Specific variable	Frequency (n)
Basic volume and productivity	Outpatient visits	40
	Inpatient admissions	37
	Number of surgeries	31
	Emergency visits/number of cases	18
	Number of births	7
Quality and adverse outcomes	Mortality rates/number of deaths	9
	Infection rates/specific adverse outcomes	6
	Quality, safety, and adverse outcomes (complications, complaints, etc.)	5
Operational efficiency and process	Average length of stay	5
	Bed occupancy rate	6
	Resource utilization and efficiency ratios (such as bed/physician ratio)	9
	Specific process and volume indicators (such as day care, laboratory, ECG tests)	19
Case severity and complexity	DRG, MEL, HDG scores	3
	Case-mix and diagnosis-based indicators (diagnostic accuracy, discharge by diagnosis)	11
Financial and institutional	Revenue	13
	Institutional and financial performance (ratios, staff satisfaction)	7
General health and accessibility	General health and access indicators (life expectancy, spending ratio, service access)	4

In the second phase of this study, a comprehensive literature review was conducted to holistically reveal the methodological diversity and analytical objectives of studies that use DEA and machine learning methods together. Within the scope of the review, a total of 48 studies published between 2020 and 2025 that address DEA-ML integration in different application areas were systematically analyzed. The studies were classified and compared in terms of the DEA models used, the integrated machine learning methods, and the application areas. This classification aims to reveal the purposes and analytical strategies with which DEA-ML integration is used in different fields such as finance, energy, environment, education, industry, and public services, not just the healthcare sector. The findings provide important clues for determining methodological approaches that can be used in hospital efficiency analyses. A summary of the studies created in this regard is presented in Table 5.

**Table 5:** Studies integrating data envelopment analysis (DEA) and machine learning (ML).

Author(s) and year	DEA methods	Machine learning methods	Application domain	Total quality score
Aydin and Yurdakul (63)	Weighted Stochastic Imprecise DEA (WSIDEA)	k-means, Hierarchical Clustering, Decision Tree, Random Forest	Country-level COVID-19 performance	7
Appiahene et al. (64)	CCR-DEA (Two-stage: Deposit & Investment)	Decision Tree (C5.0), Random Forest, Artificial Neural Network	Banking sector (Bank branch operational efficiency, Ghana)	7
Najadat et al. (65)	CCR, BCC	Ensemble Decision Trees (DECORATE)	Finance and Industrial Firms	6
Nazari et al. (66)	Super-efficiency DEA	Support Vector Machine (SVM), Artificial Neural Network (ANN), k-Nearest Neighbors (KNN), Decision Trees	Economics, Banking, and Finance	6
Mirmozaffari et al. (67)	CCR, BCC, FDH, Window DEA	k-means clustering, pre-processing filters	Cement Industry	7
Singpai and Wu (68)	CCR, BCC (VRS, output-oriented)	AutoML; Artificial Neural Network (BPNN)	Energy/Environment/Sustainability (SDGs)	7
De La Hoz et al. (69)	CCR-DEA	Random Forest, Decision Tree	Higher Education (Engineering Programs)	7
Hatamzad et al. (70)	CCR (CRS)	SVM, SVM-GA, KNN, Decision Tree, MLP, Logistic Regression	Road Maintenance/Transportation Infrastructure	7
Mirmozaffari et al. (71)	CCR, BCC, Additive DEA, Malmquist Productivity Index (MPI)	k-Nearest Neighbors, Decision Tree, Naïve Bayes, Association Rules (Apriori)	Cement Industry/Eco-efficiency/Energy & Environmental Management	5
Olanrewaju (72)	CCR	Artificial Neural Network (Multilayer Perceptron)	Energy	5
Xu et al. (73)	CCR	CART, Boosted Tree (BT), Random Forest (RF), Logistic Regression (LR)	COVID-19 response efficiency of U.S. states (healthcare system performance/public health policy)	7
Zhong et al. (74)	SBM	Neural Networks (NN), Support Vector Regression (SVR), XGBoost, Lasso	Banking and Finance	7
Zhu, Zhu and Emrouznejad (75)	CCR (CRS)	ANN (BPNN), GA-ANN (GANN), SVM, Improved SVM	Manufacturing/Industrial Firms	7
Ali and Shirazi (76)	VRS DEA (input-oriented)	Transformer-based NLP (BERT)	Environmental sustainability/E-waste policy	5
Liu et al. (77)	NDDF-based DEA	LightGBM	Green economic efficiency/environmental sustainability (prefecture-level cities, China)	5
Mirmozaffari et al. (78)	Additive DEA, CCR, BCC, Window DEA	k-NN, Decision Tree, Naïve Bayes	Pharmaceutical companies/firm efficiency (COVID-19 period)	6
Niu et al. (79)	SBM with undesirable outputs (global covariance)	Logistic Regression, SVM, BPNN, CART, Random Forest, GBDT, XGBoost, LightGBM, AdaBoost, Bagging	Low-carbon economic efficiency (regional/environmental sustainability)	7
Petridis et al. (80)	RDM, SBM	Support Vector Machines (classification)	Finance	7
Tsaples et al. (81)	Two-stage/multi-dimensional DEA (alternative optimization metric)	Machine learning–based exploratory modeling (EMA, feature exploration)	Country-level sustainability assessment (EU-28)	4
Wahyudi and Asrol (82)	CCR	Decision Trees and Artificial Neural Networks	Food Industry	5
Zarrin et al. (83)	SBM DEA (VRS, input-oriented), Bootstrapped DEA	Self-Organizing Map ANN (SOM-ANN), Multilayer Perceptron ANN (MLP-ANN)	Healthcare/Hospitals (Germany)	7
Zhang et al. (84)	SBM	Support Vector Regression, BPNN, Random Forest, XGBoost	Environmental Studies	7
Aparicio et al. (85)	Dynamic DEA, Dynamic FDH	Decision Tree-based Efficiency Analysis (EAT)	Manufacturing and Food Sectors	7
Duras et al. (86)	BCC, SCNLS-based DEA	LASSO, Elastic Net (EN), Adaptive LASSO (ALASSO)	Electricity distribution sector (Swedish DSOs)	7
Rajaprasad and Rambabu (87)	CCR	Logistic Regression, Random Forest, Decision Tree, XGBoost	Construction Project Sites/Occupational Health and Safety Performance	7
Yu and Lou (88)	CCR-DEA (input-oriented, CRS)	Projection Pursuit Regression (PPR); comparative models with ANN (BPNN) and SVR	Performance evaluation and efficiency prediction of DMUs	6
Nishtha et al. (89)	Interval DEA, CCR, BCC	Support Vector Regression (SVR)	Banking	7
Bogetoft et al. (90)	DEA (CCR, target setting)	Counterfactual analysis, bilevel optimization (XAI-based ML integration)	Banking	6
Çakır (91)	Context-dependent DEA	Adaptive Neuro-Fuzzy Inference System (ANFIS)	Transportation and Railways	7
Chivardi and Zamudio Sosa (92)	BCC (output-oriented) with bootstrapping	Random Forest	Technical efficiency of primary diabetes care (Mexico)	7
Khoubrane et al. (93)	CCR, BCC	CatBoost, LightGBM, XGBoost	Finance and Business	7
Omrani et al. (94)	DEA, COLS	Support Vector Regression (SVR), Fuzzy C-Means (FCM)	Electricity distribution companies (energy sector)	6
Perroni et al. (95)	CCR, BCC, IRS, DRS, SFA	SVM, KNN, Decision Tree, LDA, LM	Manufacturing/Energy Performance	7
Rezaee et al. (96)	CCR-DEA (CRS), multi-level efficiency frontiers	Random Forest, LIME, MOCE, MOPSO	Hospital performance evaluation and benchmarking (Iran)	7
Santamato et al. (97)	Output-oriented DEA (VRS, SHAP-weighted DEA)	Logistic Regression, k-means clustering, SHAP (XAI)	Healthcare/Hospitals (Italy–Apulia & Emilia-Romagna)	7
Shi and Zhao (98)	BCC	Random Forest, RBF-SVM, Logistic Regression	Banking and Finance	7
Zofio et al. (99)	CCR, BCC	Efficiency Analysis Trees (EAT), Convexified EAT (CEAT)	Higher Education Institutions	7
Boubaker et al. (100)	CSW-RA-DEA (Regression-based DEA)	Neural Networks, Support Vector Regression, Random Forest	Economics, Business, Manufacturing	7
Czyżewski et al. (101)	SBM-DEA (VRS), Luenberger Index (TFP)	Random Forest, GBM, XGBoost, CatBoost, LASSO	Agriculture, Food Security, Sustainable Development	7
de Oliveira et al. (102)	Super-SBM DEA (VRS)	Gradient Boosting (XGBoost)	Healthcare/Hospitals	7
Dipierro and De Witte (103)	CCR, BCC	Random Forest–based feature selection, Genetic Algorithm, Ant Colony Optimization	Higher Education Institutions (European universities)	7
Guillen et al. (104)	DEA with undesirable outputs (EATBoosting; enhanced hyperbolic DEA)	Gradient Tree Boosting (EATBoosting)	Manufacturing/PCB production	6
He et al. (105)	Three-stage SBM DEA, Global Malmquist Productivity Index	LASSO, LSTM, Double/Debiased Machine Learning (DML)	Green total factor productivity (cross-country analysis)	7
Joo (106)	Free Disposal Hull (FDH)	Efficiency Analysis Trees (EAT), Random Forest for EAT (RFEAT)	Health system efficiency of OECD countries	7
Mazandaran et al. (107)	CCR (input-oriented), Russell non-radial DEA	Artificial Neural Network (MLP)	Finance/Listed Companies	7
Soffan et al. (108)	BCC	Fuzzy C-Means, k-medoids, MLP, SVR, Random Forest, Gradient Boosting, Genetic Algorithm	Tax service offices/public sector efficiency (Indonesia)	7
Sun et al. (109)	BCC	Gradient Boosting Machine, Random Forest, SVM, ANN, AdaBoost	Telehealth efficiency/Healthcare services (U.S.)	7
Yılmaz et al. (110)	BCC	k-means clustering, CART, Random Forest	Global COVID-19 contagion control and treatment efficiency	7

Table 5 shows that DEA-ML integration is addressed in the literature through many different methodological combinations. On the DEA side, CCR and BCC models are predominantly used, but SBM, super-efficiency DEA, window analysis, dynamic DEA, and models including unwanted outputs have also gained prominence, especially in recent years. In terms of machine learning methods, Random Forest, support vector machines, artificial neural networks, and gradient boosting-based algorithms are heavily used for estimating efficiency scores, classification, clustering, variable selection, and explainability purposes. It is noteworthy that the number of studies focusing on health and hospitals is relatively limited; however, DEA-ML approaches developed in fields such as finance, energy, environment, and education include more advanced analytical tools (XAI, counterfactual analysis, target setting). This situation indicates that DEA applications in the healthcare sector are still predominantly addressed within the classical framework, and the analytical potential offered by DEA-ML integration is not sufficiently utilized. Therefore, adapting this methodological accumulation from different fields to hospital efficiency analyses presents a significant opportunity to both improve the accuracy of effectiveness measurement and produce more explainable and actionable outcomes from a policy and management perspective.

In this study, a systematic quality assessment process was applied to evaluate the methodological quality of the studies included in accordance with the PRISMA guidelines. The quality assessment criteria used were based on the checklist approach commonly used in systematic review studies and adapted to the methodological characteristics of DEA-machine learning studies, based on the framework proposed by Kitchenham (115). Seven criteria were used in the evaluation: (1) clarity of research objective, (2) transparency of dataset, (3) specification of DEA model, (4) clarity of machine learning method, (5) presence of model validation process, (6) use of performance evaluation metrics, and (7) interpretation of results. Each criterion was scored as 1 (yes) or 0 (no), and a total quality score between 0 and 7 was calculated for each study. The total quality scores for the literature included in the study are presented in Table 5. The findings indicate that the majority of studies clearly define their research objectives, present datasets in detail, and explicitly specify DEA and machine learning methods. However, some shortcomings are noteworthy, particularly in terms of model validation techniques (train-test separation, cross-validation, etc.) and the use of performance evaluation metrics. These shortcomings are particularly evident in studies published between 2020 and 2022, whereas recent studies show a more systematic and standards-compliant application of model validation processes. This indicates that the DEA and machine learning integration literature is becoming increasingly mature methodologically. Most of the studies reviewed largely meet the defined quality criteria, indicating that the literature has a strong methodological foundation.

The studies summarized in Table 5 reveal not only the diversity of methods used but also the purposes and analytical strategies employed in different contexts for DEA-ML integration. However, a table-based descriptive presentation is limited in its ability to comprehensively assess the relational structures, shared methodological patterns, and points of divergence between the studies. Therefore, Multiple Correspondence Analysis (MCA) was applied to visually and analytically reveal dominant methodological patterns, alternative approaches, and potential research gaps in the DEA-ML integration literature. To this end, the literature on DEA-ML integration was systematically reviewed, and a categorical dataset was created from studies selected according to defined inclusion criteria. To ensure the rigor and consistency of the coding process, a structured coding protocol was developed prior to analysis, including clear definitions and decision rules. All studies were assigned to a single dominant category per dimension according to their primary methodological focus, within six predefined dimensions. In studies with multiple methodological components, classification was based on the dominant analytical approach emphasized by the authors. The coding framework used for MCA is given in Table 6.

**Table 6:** Coding framework used in MCA.

Dimension	Categories
Purpose	Supervised ML (classification & prediction); Model interpretability; Clustering; Benchmarking/target setting
Integration direction	DEA → ML; ML → DEA; Simultaneous/hybrid; DEA-based ML
Application area	Healthcare; Finance; Industry; Education; Energy/Environment; Other
Data structure	Cross-sectional; Time-dependent data structure; Big data/text-based
DEA orientation	Input-oriented; Output-oriented; Unspecified/both
Target variable	DEA efficiency score; Multi-level DEA class; Non-efficiency/mixed target

The coding process was conducted independently by two researchers, and ambiguities were re-examined to ensure consistency. Inter-coder reliability was assessed using both percentage agreement and Cohen's kappa coefficient. Agreement rates ranged from 91.7% to 97.9%, while kappa values ranged from 0.817 to 0.960, indicating a very high level of agreement between coders. Coding differences were discussed and resolved, and Multiple Correspondence Analysis (MCA) was performed on the final consensus dataset.

**Figure 2:**
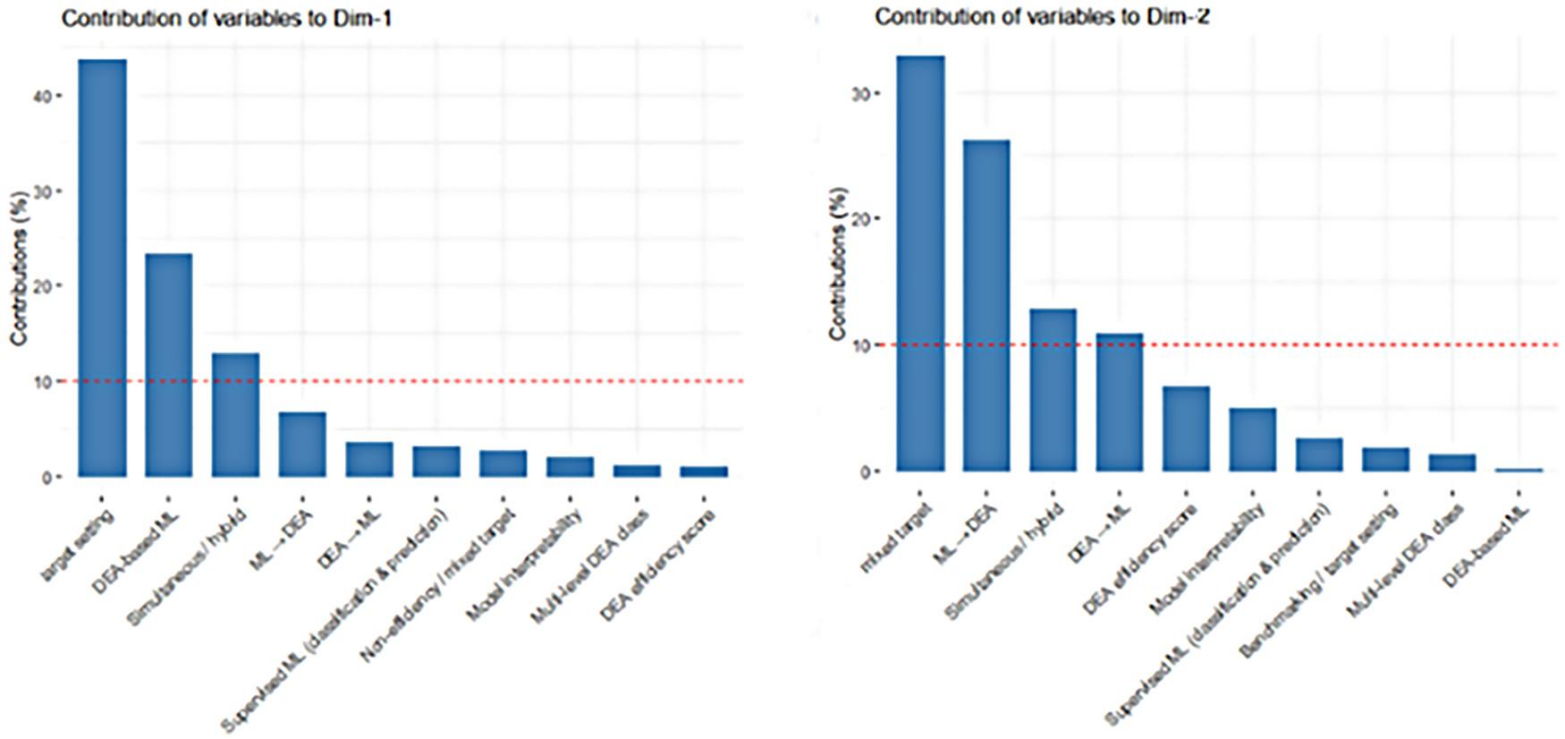
Contribution of categories to dimension 1 and dimension 2.

**Figure 3:**
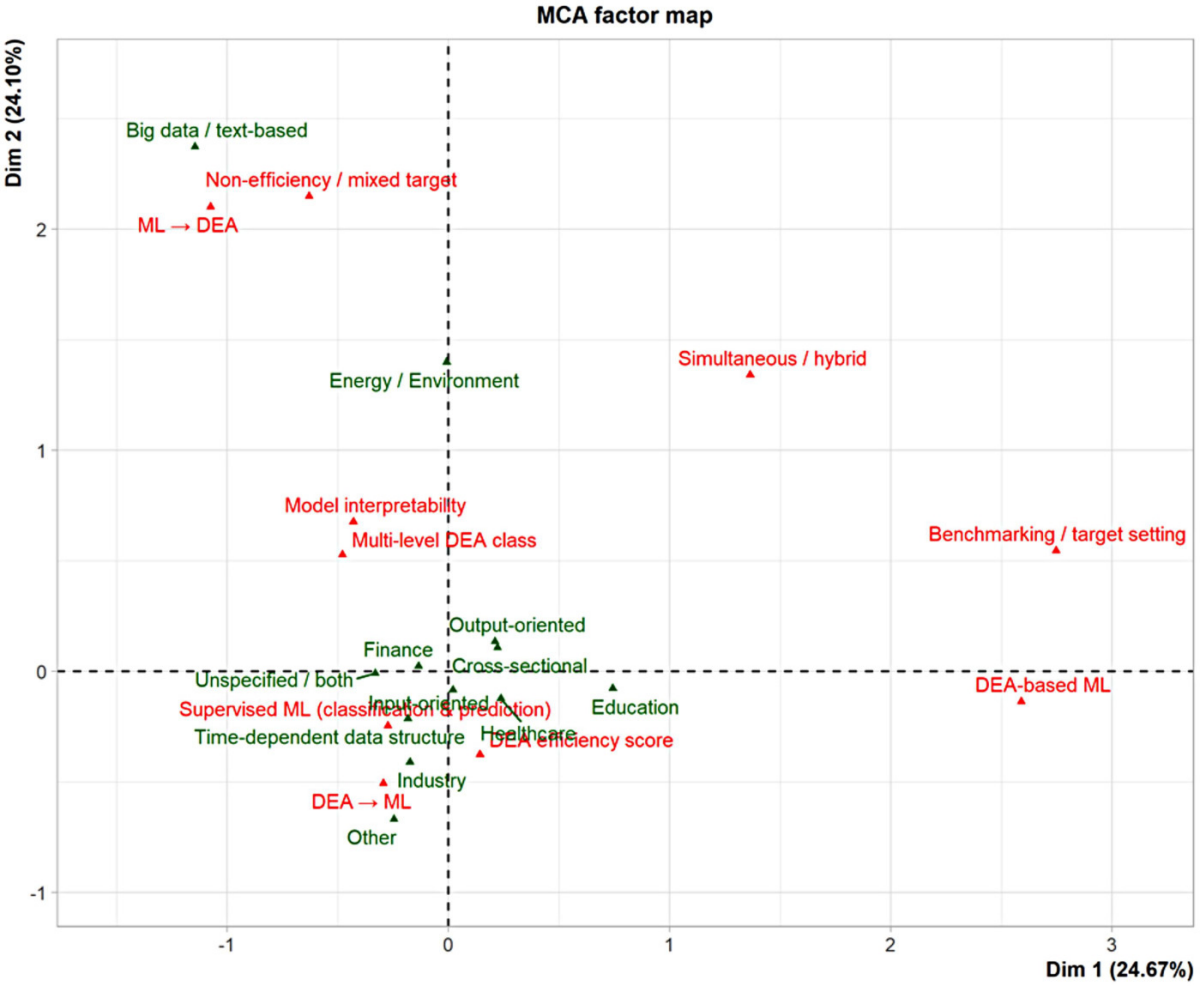
MCA Map of the DEA–ML literature.

The two-dimensional MCA map obtained from the multiple correspondence analysis, presented in Figure 3, explains approximately half of the total inertia (Dimension 1: 24.7%; Dimension 2: 24.1%). In analyses based on categorical data structures, this level of explanation indicates the presence of a meaningful and interpretable relational structure in the literature (111). To show which categories are decisive in the formation of these dimensions, the variables contributing to Dimension 1 and Dimension 2 are presented in Figure 2.

The contribution graphs presented in Figure 2 show the methodological categories that are decisive in the formation of Dimension 1 and Dimension 2 obtained within the scope of MCA. Dimension 1 is predominantly shaped by categories related to the direction of integration between DEA and machine learning and the purpose of analytical use. In contrast, Dimension 2 is primarily shaped by categories reflecting the direction of integration between DEA and machine learning and differences in target variable structures. Categories with contribution levels above the average are considered as key reference points in interpreting the relevant dimensions. To complement the graphical interpretation and provide a more detailed numerical representation, the category coordinates and contribution values are presented in Table 7.

**Table 7:** Category coordinates and contributions from multiple correspondence analysis.

Category	Dim 1	Dim 2	Contribution Dim 1 (%)	Contribution Dim 2 (%)
Supervised ML (classification & prediction)	−0.274	−0.245	3.13	2.57
Model interpretability	−0.430	0.678	1.92	4.90
Benchmarking/target setting	2.746	0.547	43.68	1.77
DEA → ML	−0.294	−0.504	3.60	10.85
ML → DEA	−1.075	2.102	6.69	26.19
Simultaneous/hybrid	1.364	1.341	12.93	12.79
DEA-based ML	2.589	−0.135	23.29	0.06
DEA efficiency score	0.142	−0.375	0.94	6.68
Multi-level DEA class	−0.480	0.529	1.07	1.33
Non-efficiency/mixed target	−0.630	2.149	2.75	32.85

When the category coordinates are examined, it is seen that the studies are distinctly differentiated according to their methodological approaches. In particular, the fact that benchmarking/target setting and DEA-based machine learning approaches are located on the positive side of Dimension 1 indicates that this dimension represents analytical approaches that are more focused on decision support and goal setting. In contrast, DEA → ML structures are located on the negative side of Dimension 1 and reflect more traditional, two-stage analysis approaches. When evaluated in terms of Dimension 2, it is observed that the ML → DEA and non-efficiency/mixed target categories are concentrated in the positive region. This indicates that the second dimension is particularly related to the integration aspect between DEA and machine learning, and the analytical use purpose of the model. When the contribution values are examined, it is seen that Dimension 1 is largely shaped by benchmarking/target setting (43.68%), while Dimension 2 is predominantly determined by the ML → DEA (26.19%) and non-efficiency/mixed target (32.85%) categories. This indicates that the contribution values reflect the extent to which each category represents the dimensional structure in a distinctive way, rather than the frequency of the categories.

Based on these numerical results, the overall relational structure among categories is visualized in the MCA factor map presented in Figure 3.

The Multiple Correspondence Analysis (MCA) results presented in Figure 3 show that the methodological preferences, analytical objectives, and application areas of studies based on DEA-ML integration are significantly differentiated under two main dimensions. The first dimension (Dim1) explains 24.7% of the total variance and mainly reflects the direction of integration between DEA and machine learning and the level of decision support-oriented use. On the positive side of this dimension are approaches that use DEA and ML simultaneously and integrally, such as benchmarking/target setting, DEA-based ML, and simultaneous/hybrid; while on the negative side are structures that are more two-stage and where DEA outputs provide input to ML models, such as DEA → ML, non-efficiency/mixed target, and model interpretability. The second dimension (Dim2) explains 24.1% of the total variance and represents the data structure and contextual complexity level of the studies. At the top of this dimension, high-dimensional, sustainability and environmental performance-focused applications such as big data/text-based, ML → DEA, and energy/environment come to the forefront; while at the bottom, studies based on more traditional, score-based and time-dependent efficiency analyses such as healthcare, efficiency score, and time-dependent data structure are concentrated.

The map shows that studies in the context of health and hospital efficiency are mostly clustered around the center and on the negative side of the second dimension. This indicates that DEA-ML studies in the health field largely rely on classical DEA models, and machine learning methods are mostly used for secondary analytical purposes such as classifying or estimating efficiency scores. In contrast, it is noteworthy that studies conducted in the fields of energy, environment, and sustainability are differentiated by more complex data structures and deeper hybrid integration strategies. Overall, the MCA findings reveal that the DEA-ML literature does not exhibit a homogeneous structure. While there is a dominant core of the literature based on supervised learning models and efficiency scores as target variables, more innovative methodological approaches are also emerging. These approaches employ DEA and ML simultaneously and integrally, often relying on mixed target variables and big data contexts. This indicates that advanced hybrid DEA–ML approaches and goal-setting-oriented analyses are still limited, particularly in the hospital efficiency literature. However, there is significant potential to adapt methodological approaches developed in other sectors to the healthcare field.

## 4 Discussion

This systematic review presents a comprehensive and multi-layered synthesis of studies using Data Envelopment Analysis (DEA) and Machine Learning (ML) methods in evaluating hospital and healthcare system performance. Covering the period 2020–2025, the analysis reveals not only the frequency of the methods used but also the analytical purposes, data structures, and integration strategies within which these methods are used together. At this point, it is important to make a clear distinction between hospital-specific findings and cross-sector methodological insights. Findings from DEA-based studies conducted in hospital and healthcare settings provide direct evidence regarding input-output structures, performance indicators, and efficiency measurement practices specific to healthcare systems. In contrast, studies conducted in other sectors are considered not as direct empirical evidence regarding hospital efficiency, but as methodological examples revealing how DEA-machine learning integration is structured in different contexts. Therefore, cross-sector findings are used to support and enrich the methodological interpretation of hospital-focused findings, rather than generating domain-specific conclusions. In this context, Multiple Correspondence Analysis (MCA), applied beyond classical table-based summaries, has allowed for a relational perspective on the methodological patterns and divergences in the literature. The MCA findings indicate that the DEA–ML literature does not exhibit a homogeneous structure. Instead, it reveals a multi-layered structure with a dominant methodological core and more innovative approaches in peripheral regions.

The majority of studies clustered in the center of the map consist of two-stage structures that use efficiency scores obtained from DEA as target variables and rely on supervised learning models (particularly Artificial Neural Networks and Support Vector Machines). This suggests that DEA is predominantly positioned in the literature as an explanatory or preprocessing tool, while ML methods mostly serve secondary analytical purposes such as classification or prediction. These findings indicate that the integration of DEA and machine learning within the scope of (RQ2) is largely shaped around two-stage structures in the literature, and that more advanced, simultaneous, and hybrid approaches remain limited.

It is noteworthy that this core structure largely overlaps with the health and hospital efficiency literature. The majority of health-focused studies included in the review prefer the BCC model, which is based on the assumption of variable scale returns; focusing on traditional capacity indicators such as bed capacity, physician and nurse numbers as inputs, and patient volume-based metrics as outputs. The use of the Malmquist Productivity Index (MPI), particularly in the post-pandemic period to track temporal change, reflects the increasing interest in dynamic efficiency analysis in health systems. However, while the integration of indicators reflecting quality and patient safety (e.g., mortality or infection rates) is increasing, inconsistencies in the use of these variables limit the production of generalizable results for value-based performance evaluations in healthcare. This situation reveals that the input and output variables used in hospital efficiency studies under (RQ1) are largely based on traditional capacity and service volume indicators; while quality and financial indicators are increasing, they are not yet consistently integrated.

The findings presented in Tables 3, 4 demonstrate that the use of quality-based output variables in DEA (machine learning) studies is shaped not only by analytical preferences but also by structural and organizational factors. Health data is considered personal health information (PHI) because it is directly related to patient care and is subject to stricter legal and ethical protection compared to other types of data. In this context, regulatory frameworks such as GDPR and HIPAA impose significant limitations on data access, sharing, and use; and mandate principles such as explicit consent, data minimization, and access control (112). However, the use of quality indicators is also closely related to the measurement difficulties arising from the nature of these variables. Quality in healthcare is a multidimensional concept encompassing multiple aspects and varying depending on patients' individual preferences and values. While this introduces a certain degree of subjectivity to quality measurement, the persistence of differences in application, even in areas with strong scientific evidence, makes the standardization of quality indicators difficult (113). Furthermore, the differences in data protection regulations and quality measurement standards between countries and health systems make it difficult to develop comparable and generalizable quality indicators. Indeed, the literature clearly emphasizes that heterogeneity in data protection practices constitutes a significant obstacle to international health system comparisons (114). In this context, observed findings should be evaluated not only in terms of methodological preferences but also in conjunction with multidimensional factors such as data access, regulatory structure, and measurement challenges.

Studies located in the peripheral regions of the MCA map represent approaches where DEA and ML are integrated more deeply and simultaneously. These studies often use mixed target variables and analyze big data or text-based structures. A significant portion of these studies focus on the fields of energy, environment, and sustainability; they utilize more advanced ML techniques such as Random Forest, boosting algorithms, and explainable artificial intelligence (XAI) tools. These findings demonstrate that, within the scope of (RQ3), DEA-machine learning integration is not limited to the healthcare sector but is being applied in different fields such as energy, environment, and finance with more advanced data structures and analytical approaches. This indicates that methodological innovations in DEA-ML integration are being adopted more rapidly in fields outside the healthcare sector, while these approaches remain limited in the healthcare sector.

In this context, the distribution of the studies examined according to their application areas also supports this observation. Of the 48 studies included in the review, 6 focus directly on hospitals and healthcare services, and this number reaches 10 when studies on healthcare system performance and COVID-19 are also included. The remaining studies are distributed across different fields such as energy, environment, finance, industry, and public services. This distribution demonstrates the widespread use of DEA-machine learning integration in various application areas, rather than a gap in the health literature. Although the relatively limited number of health-focused studies might be considered an imbalance, this does not weaken the validity of the study's findings. On the contrary, the inclusion of studies from different fields reveals the methodological diversity and analytical potential of DEA-machine learning integration more comprehensively. This cross-disciplinary perspective contributes to the identification of analytical approaches that are not yet widely used in the healthcare sector but have significant potential. Finally, when the findings are evaluated within the scope of RQ4, it is seen that the DEA-machine learning literature can be classified within dominant, transitional, and innovative methodological clusters; and that the transition between these clusters is still limited, especially in the healthcare field. These findings reveal that the integration of DEA-ML in the healthcare sector is still predominantly based on traditional, two-stage, and score-based structures; while advanced hybrid approaches focused on decision support, goal setting, and explainability are a developing research area. This methodological gap indicated by the MCA results provides an important guiding framework for future studies in healthcare. In particular, the use of real-time administrative and clinical datasets, more consistent integration of quality-oriented outcomes, and making DEA-ML models explainable to decision-makers are among the key research needs highlighted in the literature. In conclusion, this study not only summarizes current methodological trends in the hospital efficiency literature but also provides a methodological roadmap for advanced hybrid approaches applicable in healthcare by highlighting the structural and relational dimensions of DEA-ML integration through MCA. In this respect, the study makes a critical and guiding contribution to the literature on the more standardized, comparable, and policy-meaningful use of DEA and machine learning in healthcare research.

## 5 Conclusion

This systematic review comprehensively reveals how Data Envelopment Analysis (DEA) and Machine Learning (ML) methods are used in evaluating performance and efficiency in healthcare, and within which analytical strategies they are integrated. Findings covering the period 2020–2025 show that DEA continues to provide a flexible framework for efficiency analysis in healthcare.

The findings indicate that the DEA–ML literature is characterized by a dominant core based on traditional approaches, while more advanced and integrated methods remain limited. This structural divergence reveals that advanced hybrid models developed in areas such as energy, environment, and sustainability have only been reflected to a limited extent in the healthcare sector. This finding highlights a structural divergence between healthcare and other domains in terms of DEA-ML integration.

The review findings indicate that input structures used in hospital efficiency studies are increasingly focused on financial indicators, and that classical non-parametric DEA approaches are being supported by tree-based and ensemble-based machine learning methods to enhance their distinctiveness. However, it is observed that DEA models used in the literature largely concentrate around traditional BCC and CCR frameworks; physical capacity and basic human resources dominate as inputs, while service volume dominates as outputs. Although quality, patient safety, and process-based outputs have begun to be incorporated more frequently, their use remains inconsistent across studies.

Overall, this study describes how DEA and machine learning methods are positioned in healthcare research. It also identifies key methodological gaps by highlighting the structural and relational dimensions of this integration. These findings collectively underscore the need for more coherent and methodologically consistent DEA-ML applications in healthcare. In this respect, the study offers a holistic and guiding methodological framework for efficiency analysis in healthcare for academics, policymakers, and practitioners.

## 6 Future research directions

The findings of this study demonstrate certain methodological limitations in the DEA-machine learning literature, particularly in the context of healthcare and hospital efficiency. Firstly, existing studies largely rely on two-stage structures where efficiency scores obtained from DEA are used as target variables in machine learning models. Therefore, it is important for future research to address efficiency measurement and machine learning processes within integrated models rather than as separate stages.

Furthermore, it was determined that the input and output variables used in hospital efficiency studies are predominantly based on capacity and service volume, while quality and patient safety indicators are used in a limited and inconsistent manner. Therefore, future studies that systematically integrate quality-oriented and process-based indicators into models will contribute to more comprehensive performance evaluations.

Furthermore, the Multiple Correspondence Analysis results show that DEA-machine learning applications in the healthcare field are mostly concentrated around traditional approaches, whereas more advanced hybrid models are used in fields such as energy and environment. In line with this finding, adapting advanced DEA-machine learning approaches developed in other fields to hospital and healthcare system data will increase analytical depth and decision support capacity.

Finally, current studies show that machine learning methods are mostly used for classification and prediction purposes. Therefore, it is important for future research to focus on how these methods can be improved not only for prediction but also to enhance the interpretability of efficiency analyses and their usability for decision-makers.

## 7 Limitations

This study provides a comprehensive systematic review and methodological synthesis of DEA-ML integration in healthcare, but it has some limitations. First, the included studies exhibit significant diversity in terms of DEA modeling approaches, machine learning algorithms, and analytical designs. While this reflects the evolving nature of the field, it limits the direct comparability of the efficiency results obtained between the studies. Similarly, differences in the definitions of input and output variables limit comparability across studies. In particular, the inconsistent integration of quality and patient safety indicators requires a cautious interpretation of the findings. Secondly, the MCA used in this study is an exploratory method and does not allow causal inferences. MCA results are sensitive to how categorical variables are defined and do not offer causal inferences. Furthermore, the fact that DEA-ML applications in healthcare have largely gained momentum in the post-2020 period naturally limited the number of studies included in the review. These limitations, rather than weakening the study's findings, highlight the need for more standardized, transparent, and methodologically mature approaches to DEA-ML integration in healthcare. The scope of this study is limited to the years 2020–2025. This choice is related to the increasing use of DEA-machine learning integration, especially in recent years, and the emergence of methodological diversity during this period. However, excluding studies developed before this time frame is a limitation of the study. Nevertheless, since this study aims to reveal the current state of methodological trends and especially the analytical approaches that have emerged recently, its focus is limited to the recent literature. In this context, the findings reflect current methodological developments and are geared towards analyzing current trends rather than comprehensively addressing the historical development of the literature.

## Data Availability

The original contributions presented in the study are included in the article/Supplementary Material, further inquiries can be directed to the corresponding author.
